# Free Radical Scavenging, Cytotoxic and Hemolytic Activities from Leaves of *Acacia nilotica* (L.) Wild. ex. Delile subsp. *indica* (Benth.) Brenan

**DOI:** 10.1093/ecam/neq060

**Published:** 2011-06-15

**Authors:** T. Kalaivani, C. Rajasekaran, K. Suthindhiran, Lazar Mathew

**Affiliations:** School of Bio Sciences and Technology, VIT University, Vellore 632014, Tamil Nadu, India

## Abstract

Dietary intake of phytochemicals having antioxidant activity is associated with a lower risk of mortality from many diseases. Therefore, the aim of this study was to determine the free radical scavenging, cytotoxic and hemolytic activities of leaves of *Acacia nilotica* by using various methods. The results of the present study revealed that ethanol extract was the most effective and IC_50_ value was found to be 53.6 **μ**g mL^−1^ for Vero cell lines and 28.9 **μ**g mL^−1^ for Hela cell lines in cytotoxicity assays. The zone of color retention was 14.2 mm in **β**-carotene bleaching assay, which was as significant as positive control, butylated hydroxy toluene. None of the tested extracts possessed any hemolytic activity against rat and human erythrocytes revealing their cytotoxic mechanism and non-toxicity. Thus, only the ethanol extract could be considered as a potential source of anticancer and antioxidant compounds. Further phytochemical studies will be performed for specification of the biologically active principles.

## 1. Introduction

Cancer is a leading cause of death in many developed and developing countries. Although the etiology of the cancer are many, free radicals play a major role for the pathophysiological processes. These free radicals have been shown to be carcinogenic and exert their effects by various mechanisms such as causing DNA damage, altering cell-signaling pathways and modulating gene expression [1].

The free radicals are the byproducts of aerobic metabolism and are produced by endogenous sources such as mitochondrial leak, respiratory burst, enzyme reactions and auto-oxidant reactions and environmental sources such as cigarette smoke, pollutants, ultraviolet light, ionizing radiation and xenobiotics [2]. Although they play an important role in many of the physiological functions like defense, inflammation, signal transduction, cell-cell adhesion, cell proliferation transcription and apoptosis [3], their accumulation leads to pathophysiological conditions such as neuro degenerative disorders, cardiovascular diseases, diabetes and cancer [4–6].

The body has effective antioxidant defense systems that protect it from pathophysiological conditions caused by oxidative damage. The protection of the organism against oxidative stress lies not only on endogenous antioxidants but also on exogenous compounds taken in food.

Therefore, dietary supplementation is necessary that could help strengthen the antioxidant system of the body by inhibiting free radical generation and preventing chronic diseases [7, 8]. There are evidences suggesting that some edible plants as a whole or their identified ingredients with antioxidant properties have substantial protective effects on human carcinogenesis [9, 10]. Many of the plant species are already investigated in the search for novel antioxidants with potent pharmacological activities. Yet, there remains much demand to investigate more information about pharmacological potential of plant species.

Screening of medicinal plants for biologically active compounds has become a vital source of cancer-related drugs. Most of the anticancer drugs currently used in chemotherapy are cytotoxic to normal cells, leading to unwanted side effects. Therapeutically effective doses of many anticancer drugs such as anthracyclines induce oxidative stress in normal tissues like heart and brain [11]. Therefore, a search for compounds that can reduce the harmful side effects of anticancer drugs in normal tissues is necessary [12].

Complementary and alternative medicine is one of the emerging fields in health care today, especially as supportive medicine in treating diseases like cancer [13, 14]. Some modern drugs have been deduced from folklore and traditional medicines [15, 16]. Therefore, the safe traditional medicinal plants are investigated to obtain potential chemotherapeutic drugs [17]. Plant-derived natural products such as flavonoids, terpenes, alkaloids, alpha-tocopherol and carotenoids have received considerable attention in recent years due to their diverse pharmacological properties, including cytotoxic and chemo preventive effects [18, 19].

Therefore, in the present study, *Acacia nilotica*, belonging to the family Mimosaceae has been selected. The plant *A. nilotica* is a tree 5–20 m high with a dense spheric crown, stems and branches usually dark or black colored, fissured bark, grey-pinkish slash, exuding a reddish low quality gum. Leaves are found to be rich in rutin and Apigenin-6,8-*bis*-C-*β*-d-glucopyranoside [20]. It has been used by traditional healers of different regions of Chhattisgarh mainly in the treatment of various types of cancer like mouth, bone and skin. In West Africa, the bark or gum is used for cancers and tumors of ear, eye or testicles. Several studies have reported that extracts from *Acacia* species, rich in phenolics have strong antioxidant activity [21–23]. Phenolic compounds exert their protective effects by acting as blocking agents or suppressing agents in the prevention of formation of carcinogens from precursor substances [24].

In a previous study, we showed that the ethanol extract (EA) prepared from leaves of *A. nilotica* had potent antioxidant activity and was significant in comparison with all the positive controls used in our study such as quercetin, tocopherol, ascorbic acid and catechin in DPPH assay. We also reported that phenolic compounds present in EA were responsible for the antioxidant activity of the plant. Additionally, we demonstrated the possible antioxidant mechanism of the EA [25].

In continuation of the above study, further phytochemical analysis of the plant for phytosterol and lipid contents were carried out. Since phenolic compounds are responsible for the antioxidant activity, the phenolic contents were also analyzed by using HPTLC. Further investigation of other pharmacological activities such as cytotoxic and hemolytic activities of these extracts was done first time because *A. nilotica* is used traditionally to treat various types of cancer. This study reveals the *in vitro* antioxidant activity using beta-carotene bleaching and total antioxidant assays. It also establishes the cytotoxic activity of the plant on Hela (cancer) cells and on Vero (normal) cells by MTT [3-(4,5-dimethylthiazol-2-yl)-2,5-diphenyltetrazolium bromide, a tetrazole] assay. The hemolytic activity of the extracts was also performed to rule out the possible cytotoxic mechanism and to check the safety of the phytocompound thus making it suitable for the preparation of natural drugs.

## 2. Methods

### 2.1. Plant Material

The leaves of *A. nilotica* were collected from VIT University vicinity, Vellore during the month of August 2007. It was identified by Dr. G. V. S. Murthy and a voucher specimen number 1035 was deposited at Botanical Survey of India, Southern Regional Centre, TNAU campus, Coimbatore.

### 2.2. Preparation of Extracts

Air-dried *A. nilotica* leaves were packed into a soxhlet apparatus and were extracted sequentially with petroleum ether (PE), benzene (BZ), dichloromethane (DCM), chloroform (CF), Ethanol (EA) and water (AQ). The organic extracts were dried over vacuum desiccator and the solvent was removed in vacuum (40°C). The extracts were dissolved in dimethyl sulfoxide (DMSO), EA or double distilled water prior to analysis, depending upon solubility. The extracts were subjected to further analysis and all the assays were done in triplicates.

### 2.3. Phytochemical Analysis

#### 2.3.1. Qualitative Analysis

The extracts were tested to determine the presence of various phytochemicals like phytosterols, lipids, phenolic compounds, flavonoids and saponins [26].

#### 2.3.2. Quantitative Determination

The extracts were quantitatively determined for phytosterols and lipids [27, 28].

#### 2.3.3. HPTLC Analysis of Phenolic Compounds

An amount of 50 mg of the given sample extract was dissolved in ethanol and made up to 1 mL with ethanol. The solution was centrifuged and the supernatant liquid was collected. This solution was used as test solutions. For HPTLC analysis, 2.5 *μ*L of each test solution were loaded as 8 mm band length in the 5 × 10 Silica gel 60F_254_ TLC plate using Hamilton syringe and CAMAG LINOMAT 5 instrument.

The plate loaded with samples was kept in TLC twin trough developing chamber (after saturation with solvent vapor) with mobile phase (ethyl acetate : toluene : formic acid : methanol—2.3 : 3.5 : 1.2 : 0.8) and the plate was developed in the mobile phase up to 90 mm. The developed plate was dried by hot air to evaporate solvents from the plate. The plate was kept in Photo-documentation chamber (CAMAG REPROSTAR 3) and the images captured at White light, UV 254 nm. Finally, the plate was fixed in scanner stage and scanning was done at 254 nm. The peak densitogram was noted.

### 2.4. Antioxidant Assays

#### 2.4.1. Total Antioxidant Activity

The total antioxidant activity (TAA) was measured by modified method of Prieto et al. [29]. The reaction mixture of 3 mL (containing 0.6 M sulfuric acid, 28 mM sodium phosphate and 1% ammonium molybdate) was added to the different concentrations of extracts (10, 50, 100, 250 and 500 *μ*g) and kept in a water bath at 95°C for 60 min. Absorbance was recorded at 695 nm.

#### 2.4.2. *β*-Carotene Bleaching Assay

This was done by modification of established methods [30]. Linoleic acid solution (10 mL of 2 mg mL^−1^ solution in EA) and *β*-carotene solution (10 mL, 2 mg mL^−1^ solution in acetone) were added to the molten agar (10 mL, 2% solution in boiling water). The mixture was then shaken to give an orange color. The agar was then poured into Petri dishes (25 mL per dish, diameter 9 cm) and was kept in dark and left standing to allow the agar to set. Holes (4-mm diameter) were then punched into the agar, extract (1 mg) each in DMSO were transferred into the holes, and the Petri dishes were then incubated at 45°C for 4 h. A zone of color retention around the hole after incubation indicated sample with antioxidant activities. The zone diameter was also measured.

### 2.5. Cytotoxic Activity

#### 2.5.1. Cell Culture

Vero cell lines and Hela cell lines were obtained from ATCC and maintained in RPMI 1640 (Gibco, India) medium, supplemented with 10% FBS (v/v) and 100 mg l^−1^ streptomycin and 100 IU mL^−1^ penicillin (Himedia, India) at 37°C in a CO_2_ incubator with 5% CO_2_.

#### 2.5.2. Preparation of Stock Solution

The extracts were used to prepare stock using solvent (1 mg mL^−1^). The appropriate concentration of the extract was made by serial dilution with the medium.

#### 2.5.3. MTT Cell Proliferation Assay

The cytotoxic activity of the extracts (0–100 *μ*g mL^−1^) on Vero and Hela cells (1 × 10^5^ cells/well) were checked as described by modification of a method by Mosmann [31], using the CellQuanti-MTT cell viability assay kit (Bioassay Systems). The optical density was measured at 570 nm for each well on an absorbance plate reader. The wells with only culture medium or treated with 0.1% of DMSO served as control. The average of the blank controls were determined and subtracted from the absorbance values. The graph was plotted with cell viability against various concentrations of the extracts. The mean and the IC_50_ value were calculated by non-linear regression analysis using the data analysis software (Prism).

#### 2.5.4. *In Vitro* Hemolytic Assay

Hemolytic effect of different extracts on human and rat erythrocytes was evaluated by using washed erythrocytes (RBCs). For the preparation of human and rat erythrocytes, the method of Malagoli [32] was followed. Blood samples from Charles foster strain rats were collected (each weighing 130–180 g) in citrated tubes. The cells were then washed three times with 20 mM Tris-HCl containing 144 mM NaCl (pH 7.4) and a 2% erythrocyte suspension was prepared. Human erythrocytes were obtained from the peripheral blood (O positive) of a healthy volunteer. The blood was used within 24 h after bleeding and washed three times in nine volumes of sterile 0.85% NaCl saline solution. After each washing, cells were centrifuged 150 g for 5 min and the supernatant was discarded. The final pellet was diluted 1 : 9 (v/v) in sterile 0.85% NaCl saline solution and then in 1 : 24 (v/v) sterile Dulbecco's phosphate buffer saline (D-PBS), pH 7.0 containing 0.5 mM boric acid and 1 mM calcium chloride.

The hemolytic activity of the crude extract was tested [32] under *in vitro* conditions in 96-well plates. Each well received 100 *μ*L of 0.85% NaCl solution containing 10 mM CaCl_2_. The first well served as negative control containing only solvent. In the second well, 100 *μ*L of extracts of various concentrations (5–500 *μ*g mL^−1^) were added. The last well served as positive control containing 20 *μ*L of 0.1% Triton X-100 in 0.85% saline. Each well then received 100 *μ*L of a 2% suspension of rat and human erythrocytes in 0.85% saline containing 10 mM CaCl_2_. After 30-min incubation at room temperature, cells were centrifuged and the supernatant was used to measure the absorbance of the liberated hemoglobin at 540 nm. The average value was calculated from triplicate assays.

### 2.6. Statistical Analysis

All experiments were repeated in triplicate. Results were reported as Mean ± SE. The statistical significance between antioxidant activity and cytotoxicity values of the extracts was evaluated with one-way ANOVA followed by Holm-Sidak test. *P*-values less than .05 were considered statistically significant. The IC_50_ values were calculated by non-linear regression analysis using the data analysis software (Prism).

## 3. Results

### 3.1. Phytochemical Analysis

The results of the phytochemical studies showed that all the tested extracts contain flavonoids and phenols, but higher amounts were found in the EA (Table 1). In our previous study, we quantitatively determined the phenolics and flavonoids by using spectrophotometric methods and our results revealed that the ethanolic extract had higher phenolics (536.02 ± 10.9 gallic acid equivalents per gram extract) and total flavonoids (36.60 ± 1.18 quercetin equivalents per gram extract). We also found out that there was a close correlation between total phenolics and antioxidant activity.

**Figure 1:**
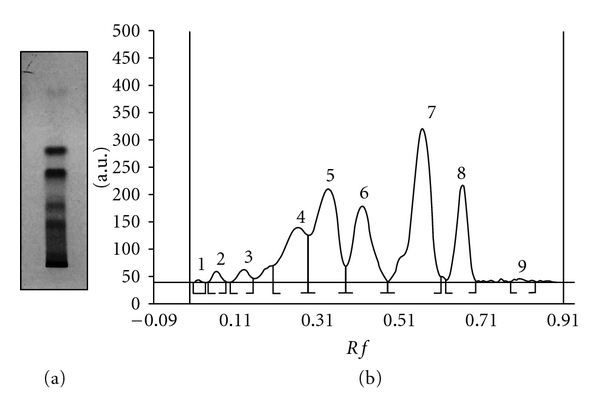
HPTLC chromatogram of EA extracts obtained from leaves of *A. nilotica* showing phenolic compounds. (a) TLC of phenolic compounds after exposure to 254 nm. (b) Densitogram of phenolic compounds showing four minor and five major peaks.

Therefore, in the present study, we made an approach to find out the HPTLC pattern of phenolic compounds. The preliminary HPTLC analysis of EA shows the presence of four minor and five major compounds (Figures 1(a) and 1(b)).

The yield, sterol and lipid contents of various extracts were determined (Table 2). This revealed that sterol content was more concentrated in DCM (142.87 ± 0.68 mg g^−1^ extract) and the lipid content in BZ (607.5 ± 3.82 mg g^−1^ extract).

### 3.2. TAA

**Figure 2:**
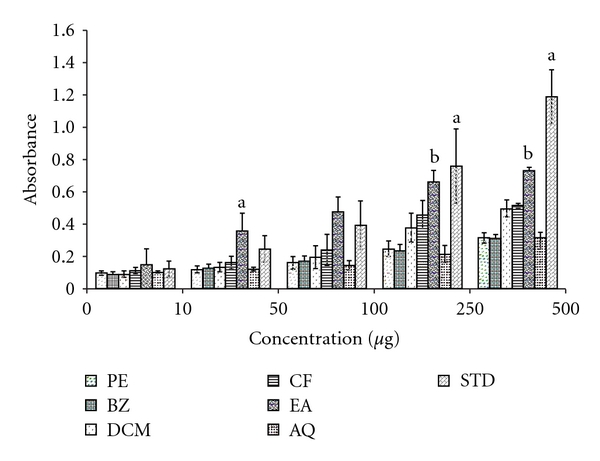
TAA of different extracts obtained from leaves of *A. nilotica*.

The assay is based on the reduction of Mo (VI) to Mo (V) by various extracts and subsequent formation of a green phosphate/Mo (V) complex at acidic pH [29]. The TAA was measured for the different extracts and for the positive control, catechin. The high absorbance values indicated that the sample possessed significant antioxidant activity. According to the results (Figure 2), the EA had significant antioxidant activities and the effects increased with increasing concentration. The order of TAA of various extracts could be seen as EA > CF > DCM > AQ > BZ > PE. There were significant differences among the extracts (*P* < .05).

### 3.3. *β*-Carotene Bleaching Assay

The antioxidant activity of the *A. nilotica* extracts were measured by the bleaching of *β*-carotene (Table 3). The positive control butylated hydroxy toluene (BHT), and all the extracts were able to inhibit the discoloration of *β*-carotene but the BHT and EA extract were significant compared to other extracts. The order was BHT > EA > AQ > CF > DCM > BZ > PE. The zone of color retention was 14.2 mm, which was close to the value of positive control BHT (15.1 mm).

### 3.4. MTT Assay

Hela cells and Vero cells were grown in 96-well assay plates and their proliferation was measured for 1 day. The assay was repeated in triplicates to ascertain whether the administration of various extracts were capable of inhibiting cellular proliferation.

The results of these experiments demonstrated that both cells were inhibited in a dose-dependent manner by various extracts, although their effects were different from one another. IC_50_ value of various extracts on both cells also exhibited distinctive patterns (Table 4). The order of cytotoxicity was EA > BZ > CF > DCM > PE > AQ for both Vero cell lines and Hela cell lines but the concentration of all the extracts required to kill the normal cells was greater than that was required for the cancer cells. The EA was the most effective, with the IC_50_ values found to be 53.6 *μ*g mL^−1^ for Vero cell lines and 28.9 *μ*g mL^−1^ for Hela cell lines.

### 3.5. Hemolytic Activity

*In vitro* hemolytic activity on rat and human erythrocytes of various extracts obtained from leaves of *A. nilotica* was performed (Table 5). The total hemolysis was obtained using 20 *μ*L of Triton X-100 (0.1%) and 1 h incubation. The IC_50_ and 95% confidence interval (95% CI) were obtained by non-linear regression analyses. IC_50_ values lower than 200 *μ*g mL^−1^ was considered active. None of the test extracts possessed any hemolytic activity against rat or human erythrocytes.

## 4. Discussion

Molecular oxygen plays a central role in the pathogenesis of various diseases. Overproduction of reactive oxygen species results in oxidative stress thereby causing cytotoxicity and cancer [12]. Cancer chemoprevention by using antioxidant approaches offers a key strategy for inhibiting, delaying or even reversal of the process of carcinogenesis. There are evidences suggesting that phenolic compounds having antioxidant activity are associated with a lower risk of mortality from many diseases like diabetes, acute hypertension, cardiovascular diseases including cancer. On the basis of this information, one of the best approaches in the search for antitumor agents from plant resources is the selection of plants based on ethno medical leads, and subsequently testing the selected plant's efficacy and safety through modern scientific methods. The study of the traditional indigenous medical practices in Amazonia and Southern Brazil has successfully introduced the ethno medicinal use of *Bidens pilosa* Linné (Asteraceae) to treat certain tumors [19].

Therefore, determination of antioxidant, cytotoxic and hemolytic activities of *A. nilotica*, which is traditionally used in cancer treatment, may identify phenolic compounds important in neutralizing free radicals and thereby reducing cell damage. There are other studies demonstrating various parts of the *A. nilotica* may exhibit antioxidant activities, but the cytotoxic and hemolytic effect of leaves of *A. nilotica* has not previously been examined. Therefore, the value of this study is the determination of antioxidant, as well as cytotoxic and hemolytic, effects of these specific extracts. Hemolytic assays were performed because compounds possessing potent antioxidant and anticancer activity may not be useful in pharmacological preparations if they possess hemolytic effect. In addition, these data also may reveal some information about the mechanism of cytotoxicity.

Phenolic compounds from many of the plants are significant protective factors against cancer. There is strong evidence for the positive correlation between cancer and dietary intake of polyphenolic compounds. Previous reports about *Acacia* species demonstrate that they are rich in polyphenolic compounds [23]. It was also reported that plant was found to be rich in gallic acid, methyl gallate and catechin. Plant-derived natural products such as phenols and flavonoids have received considerable attention in recent years due to their diverse pharmacological properties, including cytotoxic and cancer chemo preventive effects [18, 19].

HPTLC is an invaluable quality assessment tool for the evaluation of phytocompounds. It allows the efficient analysis of a large number of compounds. The preliminary HPTLC analysis of EA shows the presence of large number of phenolic compounds. Further analysis is in progress for the isolation and identification of these compounds.

TAA assay is based on the reduction of Mo (VI) to Mo (V) by various extracts and subsequent formation of a green phosphate/Mo (V) complex at acidic pH [29]. It revealed that all the extracts in this study showed an increasing antioxidant capacity with increase in concentration. However, the EA was as potent as the positive control, catechin. This could be due to the presence of phenolic compounds. Studies on the fruit extracts of citron and blood orange also revealed that phenolics are responsible for antioxidant activity [18]. This activity may be due to their reducing ability thereby donating its proton or electron to free radicals and terminating the chain reaction [33].

In the *β*-carotene bleaching assay, the biologically relevant oxidizable substrate *β*-carotene gives direct information on the protective effect of the extract [34]. Oxidation of linoleic acid produces hydrogen peroxide—derived free radicals which bleach the yellow color of *β*-carotene. The antioxidant activity (AA) was measured based on the ability of the samples to prevent the bleaching of the *β*-carotene. The presence of different antioxidants can hinder the extent of *β*-carotene-bleaching by neutralizing the linoleate-free radical and other free radicals formed in the system [35]. Accordingly, the zone of discoloration increased rapidly in samples without antioxidant whereas, in the presence of an antioxidant, they retained their color, and thus the diameter of the zone of color retention is greater. The result of this test corresponds to their free radical scavenging activity, which may be due to the presence of higher phenolics. The same was also reported in *Leucopaxillus giganteus* which also indicated the presence of higher content of phenolics with better antioxidant properties [36]. A relationship between the TAA and *β*-carotene-bleaching extent was found, suggesting that the mechanisms of action of the extracts for the antioxidant activity may be similar, related to the content of total phenolics.

Most of the anticancer drugs currently used in chemotherapy are cytotoxic to normal cells, leading to unwanted side effects. Therapeutically effective doses of many anticancer drugs produce oxidative stress in normal tissues like heart and brain [11]. Therefore, a search for compounds, which can reduce the harmful side effects of anticancer drugs in normal tissues, is necessary [12]. The integration of cancer and normal cells into the study design are therefore necessary for the detection of cytotoxic compounds.

Cytotoxic activity revealed that all extracts of the plant leaves exhibited the inhibitory effect on both cells but IC_50_ values were higher for normal cells than tumor cells. Each extract of *A. nilotica* behaved distinctly in each cell line. The distinct effects of these extracts may be due either to the phytodiversity or diverse mechanisms associated with each of the phytocompounds [37]. According to the criteria of the National Cancer Institute, a crude extract having an IC_50_ limit lower than 30 *μ*g mL^−1^ can be considered for further purification of anticancer compounds [38]. Thus, only the EA could be considered as potential sources of anticancer compounds. The potent cytotoxic activity of the EA could be due to the presence of phenolic compounds.

Phenolic compounds exert their protective effects through diverse mechanisms. They can act as blocking agents or suppressing agents thereby preventing the formation of carcinogens from precursor substances or activate a signaling cascade that activates detoxifying enzymes involved in the elimination of chemical carcinogens or induce apoptosis and cell-cycle arrest [24]. Therefore, it is necessary to find out the possible mechanism of anticancer activity. In the present study, we attempted to rule out the possible cytotoxic mechanism by using hemolytic assay. Despite the results obtained from MTT assays, none of the tested extracts possessed any hemolytic activity against rat and human erythrocytes. These data suggested the non-toxic effect of the extract thus making it suitable for the preparation of drugs involved in the treatment of various diseases. In addition, it revealed that the cytotoxic activity was not related to lytic properties or membrane instability induced by the extracts [38]. Further studies are under progress to find out the exact mechanism of its anticancer activity.

**Figure 3:**
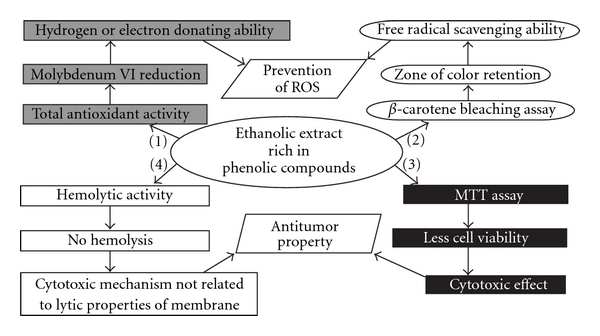
Antioxidant and anticancer potential of EA extract obtained from leaves of *A. nilotica* in different assays. Ethanol extract rich in phenolic compounds exhibit free radical scavenging activity, which was confirmed in total antioxidant activity (1) (Reduction of Molybdenum VI thereby confirming electron or hydrogen donating ability), and in *β*-carotene bleaching assay (2) (Zone of color retention indicating their free radical scavenging ability). These two activities are responsible for prevention of ROS. Ethanol extract exhibit antitumor activity, which was confirmed in MTT assay (3) (less cell viability indicating their cytotoxic effect) and hemolytic activity (4) (no lysis indicating that cytotoxic effects is not due to lytic property of the membrane).

Finally, this study revealed that EA presented more potent activity than other extracts in these assays (Figure 3). Thus, only the EA could be considered as potential sources of anticancer and antioxidant compounds. Furthermore, phytochemical studies will be done for specification of the biologically active phenolic compounds. Since, *A. nilotica* is a commonly available plant; it may represent a potential, economical therapeutic agent for cancer, and as a natural preservative, due to the antioxidant and anticancer activities.

## Figures and Tables

**Table 1:** Phytochemical analysis of *A. nilotica.*

Test	PE	BZ	DCM	CF	EA	AQ
Phytosterols	+	+	++	+	−	−
Fixed oils and fats	+	+	+	+	+	−
Phenolic compounds	+	+	+	++	+++	+
Flavonoids	+	+	+	++	+++	+
Saponins	−	−	−	−	+	+

−: not detected; +: detected (low); ++: detected (medium); +++: detected (high).

**Table 2:** Yield, phytosterol and lipid contents of various extracts from *A. nilotica.*

Extract	Yield (mg g^−1^)	Phytosterol (mg g^−1^)	Lipid (mg g^−1^)
PE	54.38	22.84 ± 0.28^(d)^	417.83 ± 1.48^(b)^
BZ	17.05	64.78 ± 0.62^(b)^	607.5 ± 3.82^(a)^
DCM	3	142.87 ± 0.68^(a)^	404.53 ± 2.37^(c)^
CF	3.6	50.94 ± 1.09^(c)^	305.9 ± 3.05^(d)^
EA	261.75	—	3.89 ± 0.07^(e)^
AQ	101.5	—	—

Means within each column with different superscript letters (a)–(e) differ significantly (*P* < .05).

**Table 3:** *β*-carotene bleaching assay of different extracts from leaves of *A. nilotica.*

Zone of color retention of *β*-carotene (mm)						
PE	BZ	DCM	CF	EA	AQ	Standard BHT
6.1 ± 0.4^(g)^	7.7 ± 0.3^(f)^	9.5 ± 0.7^(e)^	10.3 ± 0.1^(d)^	14.2 ± 0.6^(b)^	12.3 ± 0.2^(c)^	15.1 ± 0.2^(a)^

Means between columns with different superscript letters (a)–(g) differ significantly (*P* < .05).

**Table 4:** Cytotoxic activity of various extracts obtained from leaves of *A. nilotica* on Vero and Hela cell lines.

Extracts	IC_50_ (*μ*g mL^−1^)	
	Vero	Hela
PE	79.0^(e)^	48.5^(e)^
BZ	65.4^(b)^	34.1^(b)^
DCM	78.2^(d)^	43.7^(d)^
CF	70.5^(c)^	40.9^(c)^
EA	53.6^(a)^	28.9^(a)^
AQ	>100^(f)^	>100^(f)^

Means within each column with different superscript letters (a)–(f) differ significantly (*P* < .05).

**Table 5:** *In vitro* hemolytic activity of various extracts obtained from leaves of *A. nilotica* on mouse and human erythrocytes.

Extracts	Mouse-IC_50_ (*μ*g mL^−1^)	Human-IC_50_ (*μ*g mL^−1^)
PE	>500	>500
BZ	>500	>500
DCM	366	>500
CF	>500	>500
EA	478	>500
AQ	>500	>500
